# Taking the principle of the primacy of the human being seriously

**DOI:** 10.1007/s11019-021-10043-2

**Published:** 2021-07-27

**Authors:** Joanna Różyńska

**Affiliations:** Center for Bioethics and Biolaw, Faculty of Philosophy, University of Warsaw, Krakowskie Przedmiescie 3, 00-047 Warsaw, Poland

**Keywords:** Ethics of clinical research, The primacy of the human being, Declaration of Helsinki, Beneficence, Dignity, Acceptable risk

## Abstract

This paper targets an orphan topic in research ethics, namely the so called principle of the primacy of the human being, which states that the interests of the human subject should always take precedence over the interests of science and society. Although the principle occupies the central position in the majority of international ethical and legal standards for biomedical research, it has been commented in the literature mainly in passing. With a few notable exceptions, there is little in-depth discussion about the meaning and role of the principle. Several authors note that the principle is vogue, ambiguous and apparently conflicting with the accepted practice of conducting non-beneficial research on individuals unable to give consent. There are opinions that it is just “a vacuous figure of speech” and should be abandoned. This paper argues that the primacy principle is far from being “a vacuous figure of speech”, rather it should be seen as a threefold concept: a fundamental interpretative rule, a procedural rule, and a substantive rule aimed at protecting research subjects from instrumental treatment and unacceptable risks. This interpretation tracks back to the principle regulatory and normative origins in the Declaration of Helsinki of 1975, but also acknowledges changes in research ethics and practice, which took place at the turn on the twentieth and twenty-first centuries. Thus, the proposed reading of the principle is not only original, but also historically grounded and normatively fruitful. It provides a fresh and ethically rich perspective on extensively debated, but still controversial problem of an upper limit of permissible risks in non-beneficial studies.

## Introduction

Biomedical research involving human subjects, as a social practice, finds its fundamental ethical justification in the principle of social beneficence, which calls for promoting social good. The primary purpose of this social practice is to develop generalizable knowledge which shall contribute to the development of safer and more effective preventive diagnostic and therapeutic healthcare interventions or public health measures. Thus, biomedical research has as its purpose the advancement of human health and well-being—goals, which are highly valued by all societies.

Although “progress towards a world where all can enjoy optimal health and health care is crucially dependent on all kinds of research including research involving humans” (Council for International Organizations of Medical Sciences 2016, p. xii), there is a universal consensus that this goal must not be achieved at any cost. All existing guidelines and regulations on biomedical research stipulate that studies involving humans must be carried out in a way that shows respect and concern for each individual participant as well as respect for other widely accepted ethical values and principles. For example, the Declaration of Helsinki clearly states that “medical research is subject to ethical standards that promote and ensure respect for all human subjects and protect their health and rights” (World Medical Association 2013, para. 7). Guideline 1 of the International Ethical Guidelines for Health-related Research Involving Humans goes even further by stating that “although scientific and social value are the fundamental justification for undertaking research, researchers, sponsors, research ethics committees and health authorities have a moral obligation to ensure that all research is carried out in ways that uphold human rights, and respect, protect, and are fair to study participants and the communities in which the research is conducted. Scientific and social value cannot legitimate subjecting study participants or host communities to mistreatment, or injustice” (Council for International Organizations of Medical Sciences 2016, Guideline 1).

The respect and concern for the interests of individual participants are most fully manifested in the—so called—principle of the primacy of the human being (later referred to as PP). Although the relevant wording may vary, the principle states that the interests of the human being (the research subject) should take precedence over the interests of science and society. The PP is “codified” in numerous well-established international ethical guidelines and legal regulations on biomedical research involving human subjects (cf. Table 1). And many scholars consider it to be the cornerstone of modern research ethics and bioethics (e.g. Parker 2010; Human and Fluss 2001; Andorno 2013). Nevertheless, it remains unclear what the PP amounts to in the ethics of biomedical research, especially research without potential to produce direct clinical benefits to the subject. By definition, interests of participants of such studies are exposed to risks of harm only for the benefit of science and society. Thus, it may be argued that those participants are used instrumentally to advance the interests of others. i.e., they are treated in a way that violates the PP. Despite this, however, all current international ethical standards and legal regulations allow conducting non-beneficial research—even on subjects incapable of giving consent—provided additional protective requirements are met. It is difficult to reconcile these regulations with the PP, if the latter is to be interpreted as permitting only research “with a direct positive balance for research participants” (Helgesson and Eriksson 2008, p. 55), posing no net risk (Millum et al. 2013) or as requiring research to be in the participant’s best interest (Shah 2013; Piasecki et al. 2015; Waligora et al. 2018).

**Table 1:** The principle of the primacy of the human being in selected international ethical and legal standards for biomedical research involving humans

WMA Declaration of Helsinki (1975, 1983, 1989, 1996)	“Every biomedical research project involving human subjects should be preceded by careful assessment of predictable risks in comparison with foreseeable benefits to the subject or to others. Concern for the interests of the subject must always prevail over the interest of science and society” [Section: Basic principles, para. I.5] “In research on man, the interest of science and society should never take precedence over considerations related to the well-being of the subject” [Section: Non-Therapeutic Biomedical Research Involving Human Subjects (Non-Clinical Biomedical Research), para. III. 4]
WMA Declaration of Helsinki (2000, 2004)	“In medical research on human subjects, considerations related to the well-being of the human subject *should* take precedence over the interests of science and society” [Section: Introduction, para. 5]
WMA Declaration of Helsinki (2008)	“In medical research involving human subjects, the well-being of the individual research subject *must* take precedence over all other interests” [Section: Introduction, para. 6]
WMA Declaration of Helsinki (2013)	“While the primary purpose of medical research is to generate new knowledge, this goal can never take precedence over the rights and interests of individual research subjects” [Section: General Principles, para. 8]
UNESCO Universal Declaration of Bioethics and Human Rights (2005)	“The interests and welfare of the individual *should have* priority over the sole interest of science or society” [Section: Human dignity and human rights; Article 3.2]
WHO Guidelines for good clinical practice (GCP) for trials on pharmaceutical products (1995)	“Although the benefit of the results of the trial to science and society should be taken into account, the most important considerations are those related to the rights, safety, and well-being of the research subjects” [Principle 4]
ICH Harmonised Tripartite Guideline for Good Clinical Practice E6(R1) (1996)	“The rights, safety, and well-being of the trial subjects are the most important considerations and should prevail over interests of science and society” [Paragraph 2.3]
Council of Europe Convention for the Protection of Human Rights and Dignity of the Human Being with regard to the Application of Biology and Medicine: Convention on Human Rights and Biomedicine (1997)	“The interests and welfare of the human being shall prevail over the sole interest of society or science” [Chapter 1: General Provisions, Article 2—Primacy of the human being]
Council of Europe Additional Protocol to the Convention concerning Biomedical Research (2005)	“The interests and welfare of the human being participating in research shall prevail over the sole interest of society or science” [Chapter II: General Provisions, Article 3—Primacy of the human being]
EU “Clinical Trial” Directive2001/20/EC (2001)	“… the interests of the patient always prevail over those of science and society” [Article 4(i)—Clinical trials on minors; Article 5(h)—Clinical trials on incapacitated adults not able to give informed legal consent]
EU “Clinical Trial” Regulation No 536/2014 (2014)	“In a clinical trial the rights, safety, dignity and well-being of subjects should be protected and the data generated should be reliable and robust. The interests of the subjects should always take priority over all other interests” [Recital 1]

With a notable exception of papers by Gert Helgesson and Stefan Eriksson (2008, 2011), and comments made by Colin Parker (2010), Shaun Pattinson (2012), and Jan Piasecki et al. (2015), there is little in-depth discussion in the literature about the meaning of the PP or its role in the ethics of biomedical research.

Helgesson and Eriksson propose five interpretations of the PP, each one giving rise to a different moral imperative: (i) participation in research must be *en bloc* beneficial or at least neutral for the interests of individual participants; (ii) researchers must treat subjects with due respect, or as ends in themselves; in particular, they are obliged to protect subjects’ dignity, integrity, and protect them from excessive risks; (iii) studies must be conducted in accordance with certain minimum requirements regarding how participants should be treated, set by relevant guidelines and regulations; (iv) “entire system of biomedical research should leave the individual on an expected positive balance (compared with a society where there is no biomedical research on human subjects)” (Helgesson and Eriksson 2008, p. 55; cf. Litton 2008, 2012); (v) decision on whether participation in research is in the interest of an individual must be left to this individual, or—if she is unable to give consent—to her legal representative. Helgesson and Eriksson find all these interpretations semantically or logically implausible, or conflicting with other provisions of the relevant regulatory documents (versions i, ii, iv), or simply redundant (versions: ii, iii, v). They also reject the suggestion to treat the PP as a main guiding principle due to its vagueness and illegibility. In conclusion, they argue that “the primacy principle does not seem to say anything distinct; rather it seems to be a vacuous figure of speech” (2008, p. 56), and as such, it should be removed from the regulatory documents. Their opinion is not uncommon (cf. Morris 2013; Emanuel 2013; Millum et. al 2013).

At first glance, Helgesson and Eriksson’s analysis of the PP looks comprehensive and convincing. However, it suffers from a serious problem—the authors do not explain how they come up with the proposed readings of the PP. Therefore, it is questionable whether their critique hits the target, or rather attacks a straw man. To avoid this problem, a discussion on the PP ethical value, relevance or usefulness, should be preceded by a determination of a proper normative message that arises from the PP (a sentence, clause or provision “containing” the PP, to be precise). It goes without saying that not every interpretation of a normative text is justified or correct. “Interpretation shapes the content of the norm ‘trapped’ inside the text” (Barak 2007, p. 3). It is a rational process governed by certain methods and rules, by which readers (addressees) extract the normative meaning of a text from its linguistic meanings. Usually, a text has an undisputed, single linguistic and normative meaning, easy to grasp by anyone who speaks the language in question. Because language can be ambiguous, however, a text’s meaning is sometimes difficult to clarify. In these hard cases, the process of interpretation becomes critical. The PP is an evident example of such a hard case.

This paper aims at providing a justified normative interpretation of the PP. For the sake of this interpretative inquiry, the paper takes the PP seriously, i.e., it *assumes* that the presence of the PP in numerous well-established ethical guidelines and legal regulations on human biomedical research results from a rational decision of the documents’ “reasonable authors”. In other words, the article is based on the assumption that the PP is not “a vacuous figure of speech” but, rather, conveys a valuable normative message. An assumption to the contrary would make it entirely inexplicable why the PP has been kept included in so many research guidelines for over forty years. Therefore, instead of discussing an ethical justification for keeping the PP in ethical and legal regulations on human research, the article focuses on “decoding” the proper normative meaning of the PP.

The paper argues that the PP should be interpreted as a three-dimensional concept: a procedural rule, a substantive rule, and a fundamental interpretative rule. The argument proceeds in three steps. The first step tracks back to the regulatory origins of the PP in the Declaration of Helsinki (further referred to as the DoH). Particular attention is paid to the reconstruction of the PP’s original normative meaning in the context of an ethical framework of early versions of the DoH. Those historical analyses show that the PP played two distinct roles in all pre-2000 versions of the DoH, which were both ethically important and practically valuable. The second step sketches normative changes which occurred in foundations of research ethics at the turn of the twentieth and twenty-first centuries, and their influence on the PP. It is argued that those changes enriched both ethical basis and functions of the PP rather than rendering the PP useless or “empty”. Finally, the PP as a three-dimensional notion is introduced and briefly discussed.

## Regulatory and normative origins of the PP

The origins of the PP are uncertain, but—as an international principle—it is generally traced back to the first revision of the DoH adopted by the 29th World Medical Association General Assembly in Tokyo (1975). There, for the first time, the PP appeared twice in the document: first, in paragraph 5 (2nd sentence) of the section I containing “Basic Principles” for all biomedical research involving humans: “Concern for the interests of the subject must always prevail over the interest of science and society”; and second, in paragraph 4 of section III pertaining to non-therapeutic biomedical research on volunteers: “In research on man, the interest of science and society should never take precedence over considerations related to the well-being of the subject”.

Both of the 1975 formulations of the PP and their specific places within the text of the DoH remained unchanged during three subsequent revisions of the DoH of 1983, 1986, and 1996. In other words, the original PP formulations remained valid for a quarter of a century, from 1975 until 2000. It was congruent with the fact that all pre-2000 versions of the DoH shared the same internal logic, structure, and normative framework. Despite the well-founded critique (Levine 1979), all these versions maintained a “fundamental distinction” between (i) therapeutic research aimed at providing direct diagnostic or therapeutic benefit to a patient serving as a research subject; and (ii) non-therapeutic research conducted for purely scientific reasons, with no direct clinical value to a consenting participant. (Until revision in 2000, the DoH did not permit non-therapeutic research on individuals unable to give consent). This distinction governed the internal composition of the document: before 2000, the DoH comprised of “Introduction” and three sections: I—“Basic principles”; II—“Medical research combined with professional/clinical care (clinical research)”; and III—“Non-therapeutic bioresearch involving human subjects (non-clinical biomedical research)”.

Moreover, all pre-2000 versions of the DoH consequently endorsed an ethical perspective on biomedical research called either “similarity position” (Miller and Brody 2003) or “therapeutic orientation” (Miller and Rosenstein 2003). The similarity position assumes that the ethics of researcher emanates from the ethics of physician and, as such, rests on “the same moral considerations that underlie the ethics of therapeutic medicine” (Miller and Brody 2003, p. 20; cf. Miller and Weijer 2006a, 2006b, 2007). Thus, similarly to the clinical practice, the research practice is primarily governed by the principle of therapeutic beneficence (incorporating maleficence) central to medical ethics, and it is only secondarily governed by other ethical considerations, such as the respect for autonomy. The principle of therapeutic beneficence binds the physician-researcher with a fiduciary duty to promote and protect the health interests of their patients serving as research subjects. It allows exposing patients-subjects only to risks that are justified by potential therapeutic benefits for those subjects. These fundamental moral imperatives were reinforced in all pre-2000 versions of the DoH by famous quotes: one from the WMA Declaration of Geneva: “The health of my patient will be my first consideration”, and another from the International Code of Medical Ethics: “Any act or advice which could weaken physical or mental resistance of a human being may be used only in his interest” (in the 1964 and 1975 versions of the DoH), which was replaced—in all successive versions—by “A physician shall act only in the patient's interest when providing medical care which might have the effect of weakening the physical and mental condition of the patient” (since the 2004 revision the second part of the provision, staring with “which”, has not been cited).

The therapeutic orientation of pre-2000 versions of the DoH defined an ethical horizon for the conduct of therapeutic research “in which the aim is essentially diagnostic or therapeutic for a patient” (“Introduction”). The orientation was reflected in all paragraphs of section II of pre-2000 versions of the DoH, especially in paragraph II.6, which allowed the physician-researcher to combine medical research with professional care “only to the extent that medical research is justified by its potential diagnostic or therapeutic value for the patient”. It constituted a normative basis for the DoH position on the ethical use of controls treatments, including placebo.

Until its revision in 1996, the DoH had stipulated that “in any medical study, every patient—including those of a control group, if any—should be assured of the best proven diagnostic and therapeutic method” (para. II.3). The 1996 DoH was the very first version to directly mention the use of placebo control by adding the following sentence: “This does not exclude the use of inert placebo in studies where no proven diagnostic or therapeutic method exists” (para. II.3). Thus, the 1996 DoH *expressis verbis* ruled out the use of placebo whenever proven method existed. This ethical position regarding placebo-controls—called by Emanuel & Miller “active control orthodoxy” (2001)—was heavily criticized for banning scientifically valuable studies and impeding medical progress (Temple and Ellenberg 2000; Ellenberg and Temple 2000). It was, however, fully consistent with the therapeutic orientation of the early versions of the DoH.

The primary duty of the physician-researcher to act in the best medical interest of the patient-subject leaves a narrow ambit for randomizing interventions in clinical trials in general, and conducting placebo-controlled trials in particular. This narrow ambit has been defined in bioethics literature by the concept of equipoise. Although the concept still raises many controversies (Freedman 1987; Gifford 1995; Karlawish and Lantos 1997; Aschcroft 1999; Veatch 2002, 2007; Miller and Weijer 2003, 2007; Miller and Brody 2003, 2007; Jansen 2005; London 2007; Djulbegovic 2007; Shamoo 2008; Joffe and Miller 2012; Kimmelman 2012; Hey et. al. 2017), in the most commonly accepted formulation, developed by Benjamin Freedman, the requirement of “clinical equipoise” is satisfied when there is “an honest, professional disagreement among expert clinicians about the preferred treatment” (Freedman 1987, p. 144). In other words, the clinical equipoise obtains when there is no conclusive evidence that the risk–benefit profile of an investigational drug (or other tested intervention) is superior or inferior to the risk–benefit profile of a standard treatment, which the patient would otherwise be offered (if such a treatment exists). Only in the case of genuine scientific uncertainty about comparative clinical merits of each arm of the study, the physician-researcher may legitimately offer trial enrolment to her patients without violating her primary therapeutic obligation. The concept of clinical equipoise necessarily implies that the use of placebo controls is unacceptable in all situations where a proven effective treatment exists. Such a use of placebo violates clinical equipoise because the placebo is known to be inferior to the standard treatment. The 1996 DoH reflected this normative logic (although it did not refer to the term “clinical equipoise” or alike).

While the therapeutic orientation seemed to provide an almost “natural” ethical framework for therapeutic research in all pre-2000 versions of the DoH, it did not constitute an obvious ethical foundation for the conduct of non-therapeutic research. The principle of therapeutic beneficence could have appeared appropriate to govern relations between researchers *qua* physicians and subjects *qua* patients, i.e., individuals, who because of illness and the power of medical profession, come to the physicians-researchers for expert advice and (experimental) treatment. Nevertheless, it seemed inadequate to govern the practice of non-therapeutic research, which—by definition—do not offer any prospect of direct health benefits to subjects (even if they are sick), and often involve healthy volunteers, who do not need or want any therapeutic intervention.

Obviously, all pre-2000 versions of the DoH admitted the difference. They distinguished between therapeutic research and purely scientific research. Additionally, they referred to participants of the latter as “person[s] subjected to the research” or “person[s] on whom biomedical research in carried out”, rather than “patients”. However, the recognized difference between therapeutic and non-therapeutic studies did not lead to the abandonment of the physician-researcher duty of care for the participants of non-therapeutic research. To the contrary, all pre-2000 versions of the DoH *expressis verbis* extended the principle of therapeutic beneficence, however only in its protective aspect, equally to subjects of non-therapeutic research.

All pre-2000 versions of the DoH imposed on the physician-researcher a duty to “remain a protector of the life and health” of every subject of non-therapeutic research (para. III.1) and a duty to terminate the study which could be harmful for the individual (para. III.3). By those two paragraphs physicians-researchers were obliged to protect the most fundamental interests of individuals participating in non-therapeutic research, namely their life and health, although—from a “technical” point of view—participants of that research were not their patients, and therefore were not covered by the protective shield of the moral imperative “the health of my patient will be my first consideration”. It must be stressed, however, that the strength and the scope of those obligations must have had been mitigated by the requirement to conduct non-therapeutic research on volunteers (para. III.2.), i.e., individuals able to give or refuse consent for research. Thus, physicians-researchers were bound by two potentially conflicting duties: the beneficence-based duty to protect life and health of research participants and the duty to respect individuals’ autonomous decisions regarding their participation in research. As I will show later on, one of the normative roles of the PP in the pre-2000 DoH was to navigate researchers between the *Scylla* of unjustified paternalism and the *Charybdis* of insufficient concern for welfare of participants of non-therapeutic research.

The above description of the regulatory origins of the PP and its normative context is far from being complete. Nevertheless, it is sufficient for further analyses. It shows that the PP originated in a specific ethical framework of the 1975 DoH built upon the principle of therapeutic beneficence. It furthest shows that the PP remained unchanged in all pre-2000 versions of the DoH, and that it has been treated seriously. There is no conceivable reason why the PP would have been kept repeated (in two separate places), had the drafters of the 1975–1996 revisions and the WMA members considered one or both of the PP formulations meaningless or redundant. Finally, the preceding description suggests that original normative functions of the PP were related to the therapeutic orientation of the early versions of the DoH.

The next two sections will reconstruct the meaning of the original 1975 formulations of the PP in accordance with basic tenants of a textualism—a prominent method of interpretation in common law jurisdictions (Brannon 2018), and—what is even more important—an interpretive paradigm factually prevailing in modern international law (Zarbiyev 2015, cf. Ammann 2020). Clearly, the DoH is not a legally binding document issued by a national or regional legislator; nor it is a legally binding treaty between states. But, despite this, it shares certain common important features with any statute or international treaty that makes a *per analogiam* application of the textual method of interpretation justified: (i) the DoH is a written normative document prescribing (ethical) norms, rules, and standards for a certain type of activity; (ii) it has a quasi-legal structure and a level of formalization (the text is organized into sections and numbered paragraphs; language and formulations follow basic requirements of legislative technique); (iii) it is a public and international document, meaning it has been produced by a well-recognized international organization, namely the WMA, in accordance with its mandate, for public distribution and access; moreover, it is addressed to a very broad category of subjects—physicians worldwide (and since 2000 revision of the DoH, also to other participants in medical research involving human subjects); (iv) it was subject to multi-stakeholders’ negotiations and approval in accordance with a procedure defined by the WMA’s Constitution and bylaws.

Textualism is not a unitary or uncontroversial approach (Nelson 2005; Grove 2020). Nevertheless, it is an attractive interpretative tool for the DoH, at least, for two reasons. First, all textualists focus on reconstructing objective meaning of a text which shall be evident from the document itself, instead of attempting to determine its purpose or actual intent of the drafters, unless—of course—the intent is clearly stated in the text (Brannon 2018). They focus on the document’s wording and its structure “as it is”, because it is that text that survived—often long, complicated, and deeply political—bargaining process that produced it. This textual method is based on an assumption that users (addressees) of the document usually have no access to nor the expertise to understand the historical background and circumstances which led to its adoption. And this assumption fits more than well with the reality of the readers’ position in relation to the DoH. Researchers do read and analyze the words of the DoH; they do not delve into reports of drafting committees or minutes of WMA discussions over the DoH projects.

Second, all textualists focus on linguistic conventions (as to vocabulary, grammar, etc.) that prevailed at the time of the adoption of the text. They start with the ordinary (plain, normal, customary, natural) meaning/usage of the words, and presume that these words should be given the same meaning that they would have in an everyday language, unless the text includes an explicit definition, that varies from the term’s ordinary meaning, or where there is an evidence that the term has a specialized meaning. Textualists are not literalists, however. They interpret words of a text in the light of the sematic and structural context of the text taken as a whole. Thus, they look at a given text holistically, they analyze its structure, and “hear the words as they would sound in the mind of a skilled, objectively reasonable user of words”, familiar with relevant social and communicative practices (qtd. in Brannon 2018, p. 14). To make sense of a normative document they rely on common conventions of language use, which they give a more formal representation in the so-called “canons of construction”. Generally, scholars divide the canons into two groups: (a) semantic canons, which reflect ordinary semantic, syntactic, and pragmatic rules of language use as accepted in society at large, and (b) substantive canons, which relate specifically to the interpretation of legal acts and statutes (Brannon 2018; Nelson 2005). For the sake of this analysis, only the most widely accepted of semantic canons will be taken into account. Some textualists go further in their understanding of the relevant interpretative context, rightly assuming that language is a cultural phenomenon and cannot be interpreted in cultural isolation. These—so called “flexible” textualists—allow interpreters to reach beyond the sematic context of the text itself, and “to make sense of the […] language by looking at social and policy context, normative values, and the practical consequences” (Grove 2020, p. 286). This approach fits particularly well with interpreting ethical documents, therefore it will be applied in this paper.

## Problems with the wording of the original PPs

As noted above, the PP appeared twice in all pre-2000 versions of the DoH. The PP as expressed in paragraph I.5 (2d sentence) read: “Concern for the interests of the subject must always prevail over the interest of science and society” (further referred to as the PP1). While the PP contained in paragraph III.4 stated: “In research on man, the interest of science and society should never take precedence over considerations related to the well-being of the subject” (further referred to as the PP2). Just from a quick read, it is evident that PP1 and PP2 were not identical and both suffered from serious interpretative problems. From the semantical and grammatical point of view, both sentences outlining the PP were drafted poorly.

First, they required weighting concerns for the interests or considerations related to the well-being of X against the interests of Y. But concern for, consideration and interest are distinct entities. “Concern for” stands for a certain proactive attitude (often combined with a certain emotional response) towards someone of something; “consideration” is a term for an act of thinking about something carefully; and “interest” refers to something that brings advantages to or affects someone or something. Each of these phenomena belongs to a different category. Therefore, they are incommensurable and cannot be straightforwardly weighed against each other.

Second, the use of the singular in expressions “the interests of *the subject*” or “the well-being of *the subject*” created an ambiguity as to whether the obligation to care for the participant’s interests applied to all participants of a research project taken as a group, or to every individual subject separately. A classical rule of interpretation called “number canon” states that, in general, the singular includes the plural, and vice versa (Brannon 2018, p. 55). However, if the obligation were to read as applying to all subjects taken as a group, a high concern for the interests of one sub-group of the participants could have compensated a low concern for the interests of another sub-group. On the other hand, if the obligation were to be interpreted as referring to every individual research subject, an explanation would be needed on how to reconcile the PP with research practice that always exposes participants’ interests to some risks only for the benefit of others. Undoubtedly, this is an essential challenge for anyone who wants to take the PP seriously.

Third, it was unclear whether the drafters of pre-2000 versions of the DoH treated “interests” and “well-being” of the subject as synonyms, or not; and whether they purposefully made use of different sentence structures and different verbs in paragraph I.5 (“must always prevail over”), and in paragraph III.4 (“should never take precedence over”), or whether the particular choice of words was a simple byproduct of a difficult and imperfect drafting process. The latter explanation seems probable. As one author of the 1975 revision noted, the revised version of the DoH was adopted “after much political turmoil, which is rather the rule than the exception in such political surroundings” (Riis 2003, p. 18). Still, under the textualist approach and, in particular, one of its rules of interpretation, called “presumption of consistent usage canon”, identical words used in different parts of the same text should be interpreted as having the same meaning; and conversely, difference in terms suggests a difference in meaning (Brannon 2018, p. 58). Thus, it is justified to assume that “interests” and “well-being” had different meanings. Alas, it is not easy to determine definitions and mutual semantic relations of the terms, especially as they stood over fifty years ago. Even today, none of those words has a well-defined, everyday meaning. Dictionaries often treat them as synonymous. And there are no universally accepted scientific definitions of these concepts. Contrary, there are a variety of theories of well-being and accounts of human interests in the literature (cf. Feinberg 1984; Sumner 1996; Fletcher 2016a, 2016b). Thus, the application of the primary interpretative rule of the “ordinary meaning canon”—which states that words should be given their common meanings, unless there is an indication that they have a technical sense (Brannon 2018, p. 57)—results in more questions than answers.

All those uncertainties stemming from the original wording of the PPs added to the confusion about the right interpretation of ethical standards laid down in the pre-2000 versions of the DoH. It needs to be noted, however, that the majority of those problems have been already fixed, although the WMA had needed more than thirty years to do that. The interpretative dilemmas connected with the grammatical and semantical differences between the PP1 and the PP2 vanished with the 2000 revision of the DoH, which reduced the PP to a single provision: “In medical research on human subjects, considerations related to the well-being of the human subject should take precedence over the interests of science and society (para. 5).” The categorical errors and ambiguities concerning the term “the subject” were removed during the sixth revision of the DoH in 2008, when the PP got the following format: “well-being of the individual research subject must take precedence over all other interests” (para. 6).

## Normative roles of the original PPs

As already noted, in all pre-2000 versions of the DoH the PP was spelled out in two separate paragraphs—PP1 and PP2. Did the PP1 and the PP2 serve the same or different normative functions? A widely accepted interpretative canon called “rule against surplusage” requires giving each provision of a normative text an independent, no redundant or superfluous meaning (Brannon 2018, p. 59). In other words, if a reading of a provision makes the provision redundant while another reading would avoid the redundancy, the latter reading is preferred. An initial application of this canon suggests that the PP1 and the PP2 had separate meanings and roles. This section argues that the PP1 was a procedural rule, while the PP2 was a substantive rule protecting individuals participating in purely scientific research against instrumental treatment and unacceptable risks.

### The PP as a rule of procedure

In all pre-2000 versions of the DoH the PP1 was expressed in section “Basic Principles”. However, it was not singled out as a separate ethical principle for research on humans. It was buried in about halfway down the documents, in provisions setting general requirements for research risks and potential benefits—paragraphs I.4 and I.5. Paragraph I.4 spelled out the main substantive principle regarding the acceptable risk–benefit ratio in research, namely the proportionality requirement. It read: “Biomedical research involving human subjects cannot legitimately be carried out unless the importance of the objective is in proportion to the inherent risk to the subject”. Paragraph I.5 contained procedural requirements regarding the process of risk–benefit assessment. The PP1 was expressed in the second sentence of this paragraph I.5 which stipulated: “Every biomedical research project involving human subjects should be preceded by careful assessment of predictable risks in comparison with foreseeable benefits to the subject or to others. Concern for the interests of the subject must always prevail over the interest of science and society”.

The localization of the PP1 in paragraph I.5 is crucial for its interpretation. Since the PP1 was neither singled out in a separate paragraph nor added to the clause setting the proportionality requirement, instead it was expressed by the paragraph on the process of assessing research risks and benefits, it should be interpreted as setting an additional requirement applicable to the process. Thus, it seems justified to claim that the PP1 was primary a rule of procedure: in every process of research risk–benefit assessment possible negative and positive effects of the research on the participant’s interests must be given the first and the most serious consideration, when weighing the different interests at stake, without being unequivocally overriding.

The PP1 interpreted in such a way constituted a purely procedural requirement that did not determine the outcome of the assessment. It was addressed to researchers and primarily to research ethics committees. Given the 50 years of bioethics and biomedical law debates on the role, organization, and functioning of research ethics committees, it may be difficult now to step back and fully understand the significance of the PP1. However, it must be remembered that one of the most important developments of the 1975 revision of the DoH—beside the PP—was the introduction of the requirement that every protocol of research involving humans be submitted to an independent committee for review (paragraph I.2). These newly established bodies needed the necessary substantive and procedural guidance on how to evaluate research protocols. The PP1 provided such a help. Although it was relatively laconic, the PP1 obliged researchers and research ethics committees to carefully identify and assess risks and expected benefits (if any) to the interests of the participant, and to carefully weigh them against the interests of science and society.

### The PP as a substantive rule

Differently from the PP1, which applied to all biomedical research involving human subjects (i.e., therapeutic research and non-therapeutic studies), the PP2 was limited only to the non-therapeutic studies. As mentioned previously, if the PP2 were to be interpreted also as a rule of procedure, it would be evidently redundant. Thus, the “rule against surplusage” provides support for a claim, which will be defended in this section, that the PP2 was a substantive rule protecting individuals participating in purely scientific research.

In all pre-2000 versions of the DoH, the PP2 was spelled out in the very last paragraph of the section pertaining to non-therapeutic research, which was also the final paragraph of the DoH. This place of the PP2 is important for its interpretation. Since the PP2 was listed as the fourth out of the four ethical principles for conducting non-therapeutic research, it is justified to presume that it did not introduce an entirely new content of pivotal importance (“presumption against hiding elephants in mouse holes”; Brannon 2018, p. 62). Instead, the PP2 should rather be interpreted against the normative background stemming from preceding paragraphs of the section referring to the principle of therapeutic-protective beneficence (para. III 1., III. 3), and the principle of respect for subjects’ autonomy (para. III.2). Each of those principles drew ethical limits to risks to which competent subjects of non-therapeutic research could have been exposed, even in highly valuable projects. In other words, they imposed constrains on the general requirement of proportionality of risk and potential benefits of research (para. I.4 & 1.7) governed by logic of utilitarian cost–benefit calculus. However, the risk limits set by each of the principles were not necessarily convergent. It is widely recognized that the duty to respect subjects’ decisions may conflict with the duty to protect the volunteers’ health, and it is not always clear which of them should triumph over the other. The PP2 should be interpreted against this background. The PP2 reminded researchers and research ethics committees that “In research on man, the interest of science and society should never take precedence over considerations related to the well-being of the subject” (para. III.4). At the same time, by referring to the broader term “well-being” instead of “health and life”, the PP2 also suggested how those potentially conflicting duties should be balanced against each other.

In the most general sense, the principle of respect for autonomy protects and promotes an individual’s capacity to choose according to her preferences, beliefs, and value commitments—i.e., according to her personal view of what is valuable in human life—by obliging others to respect her right to make her own decisions (Łuków and Różyńska 2015). By virtue of this right an individual can pursue her personal interests in realizing particular life goals, plan, and projects, which are an expression of her personal views on what constitutes human well-being, even at cost of putting her more basic interests, such as her health or even life, at risk. For many volunteers, participation in non-therapeutic research is not only an opportunity to earn “quick and easy money” (Abadie 2010, p. 6), but also an occasion to promote truly important personal interests, e.g., contributing to the development of medical sciences, demonstrating solidarity with one’s community or people fighting against a particular disease, paying a debt of gratitude for the previously received medical care and assistance. Thus, the commonly accepted view is that competent and well-informed subjects “do not need protection from their ability to choose to take on reasonable risks when there are important and meaningful reasons, from an individual’s point of view, even if those reasons are unrelated to one’s best medical interests” (Litton & Miller 2005, p. 569). However, as we all know, the devil is in the details—in that case in “reasonable risks”.

Undeniably, the right to self-determination is not absolute. All societies impose some ethical and legal limits on that right, particularly on the individual’s freedom to dispose of her life, body, and health. The rationale is usually paternalistic, although other moral considerations are often in play as well (e.g., moralism, prevention of harm to others, prevention of offence to others, considerations of social utility, justice or fairness). The limits imposed on an individual’s freedom may assume direct and indirect forms (Różyńska 2015). In the latter, an individual’s liberty-right to freely dispose her life, body, and health is limited by certain negative or positive obligations imposed on others which the latter must fulfill regardless of the will of the individual in question. Professional duties of physicians-researchers toward patients-subjects are excellent examples of such indirect limitations. Under the pre-2000 DoH normative framework for non-therapeutic studies, the physician-researcher had duties to protect the subject’s life and health (para. III.1, 3) and to take every precaution to “minimize the impact of the study on the subject’s physical and mental integrity and on the personality of subject” (para. I.6), even against the subject’s will. As already explained, those duties found basic ethical justification in the principle of therapeutic beneficence. They set an upper limit of reasonable risk, which a competent and willing individual could have been exposed to in non-therapeutic research. By the same token, these duties limited the individual’s right to promote her personal interests in a way that posed real treat to her life or health (physical or mental integrity).

Assuming that the presented analysis is correct, it leads to the conclusion that the PP2 was the following substantive protective rule: no matter how high the potential social value of the research is and no matter how favorable risk–benefit profile of the research is, the non-therapeutic research must not (i) exploit competent participants by exposing them to research risk without their free and informed consent; and (ii) put them at significant risk of death or other serious and irreversible physical or mental damage, even if the subjects have given informed consent.

## The PP and the shift in the ethical framework for biomedical research

The previous analysis focused on the PP in pre-2000 versions of the DoH. This section offers a very brief overview of major normative changes that occurred at the turn of the twentieth and twenty-first centuries in the ethical foundations of the DoH, and in the ethics of biomedical research on humans in general. This section investigates as well the implications of those changes for the interpretation of the PP.

By 2000, the DoH has risen in prominence as the guiding document for the ethics of biomedical research involving human subjects (Carson et al. 2004). It has been widely endorsed, implicitly or explicitly, as one of the cornerstones of international and national instruments regulating research on humans (Human and Fluss 2001). In parallel, in the mid-1990s heated debates over the standard of care owed to participants during and after research, especially those conducted in developing countries, and the ethics of placebo controlled trials started (Rothman and Michels 1994; Freedman et al., 1996a, 1996b; Angell 1997; Lurie and Wolfe 1997; Annas and Grodin 1998; Ellenberg and Temple 2000; Temple and Ellenberg 2000). Those debates revealed that the ethical paradigm of similarity position, and its “logical offspring”—clinical equipoise and “active control orthodoxy”—were not universally shared by academic ethicists, researchers (sponsors), and the relevant public authorities and policy-making bodies. To the contrary, they were rather a minority view (Lie et al. 2004). By the end of the 1990s the necessity for a change of the DoH had become evident also for the WMA. Accordingly, in 2000, after over two years of heated discussions, the 52nd WMA General Assembly adopted the fifth revision of the DoH, which proved to be the most far reaching and controversial modification of the document to date (Carlson et al. 2004; Kuroyanagi 2009). It resulted in a major normative and logical re-framing of the DoH as well as complete restructuration of the text. There is no need or space to discuss every aspect of the introduced modifications. It is, however, necessary to note that, although the references to the Declaration of Geneva and the International Code of Medical Ethics remained intact, the 1964–1996 distinction between therapeutic and non-therapeutic research was dropped. The DoH got a new internal three-part structure: “Introduction”, “Basic principles for all medical research”, and “Additional principles for medical research combined with medical care”. There was no longer a separate section dealing with non-therapeutic research. Such studies were subject to ethical standards for all medical research; now therapeutic research was awarded an additional regulation. In consequence, the 1975–1996 wording and the location of the PPs were modified. The PP was no longer mentioned twice in the 2000 DoH, nor in two separate paragraphs. It was “codified” in paragraph 5 of the introductory section as one of the top ethical considerations regarding research on humans. This is the textual and normative position that the PP has been occupying since then in the DoH (2008, 2013) and various other instruments setting standards for biomedical research involving humans.

It is also important to note that, despite major amendments proposed during the DoH review process, the 2000 revision did not introduce any significant changes in the overall ethical guidelines for placebo use (Kuroyanagi 2009). However, in response to the growing criticism against the “active control orthodoxy” of the DoH, in 2002 the WMA made an unprecedented move and added “Note of clarification” to the placebo-regarding paragraph. The “Note” enumerated two scenarios, in which a placebo-controlled trial could be ethically acceptable, even if proven therapy was available: (i) where the use of placebo control was necessary for compelling and scientifically sound methodological reasons; or (ii) where the trial investigated a method for a minor condition and “the patients who receive placebo will not be subject to any additional risk of serious or irreversible harm”. Evidently, the Note did not clarify, but did significantly modify requirements for the use of placebo controls. With the added “Note”, the DoH abandoned “active control orthodoxy” and moved to the “middle ground” position (Emanuel and Miller 2001), which at that time, was already adopted by many international standards as well as numerous advisory and regulatory bodies (Lie et al. 2004). Thus, the DoH was no longer entirely faithful to the therapeutic orientation and the principle of clinical equipoise. Crucial cracks appeared in its normative foundations as well as in the ethical authority of the DoH guidelines.

Those (and other) ethical inconsistencies in the 2000 DoH mirrored the ongoing discussions on the need to revise ethical foundations of biomedical research involving human subjects. The debates were stimulated by rapid changes taking place both in research practice and research governance (e.g., growing patients’ activism and engagement in research, commercialization and globalization of research, local communities involvement in research, increasing regulatory demands for randomized double blind placebo control studies). All those new phenomena undermined theoretical and practical accuracy of the similarity position as “the reigning paradigm in the ethics of clinical trials” (Miller and Brody 2003, p. 24). Due to space limit, in what follows, three main “revolutionary” tendencies will be briefly discussed.

### Beyond beneficence and respect for autonomy

At the end of the twentieth century, the similarity position, with its focus on the therapeutic obligation of a physician-researcher towards an individual research subject, turned out to be too narrow to embrace important ethical and social commitments represented by multiple facets of the principle of justice.

In the last two decades of the last century, the focus of research ethics and research oversight shifted from the protection against research to considerations regarding the just conduct of and access to research (cf. Emanuel and Grady 2006). Participation in research was no longer perceived as inherently dangerous, harmful or exploitive. It began to be viewed as a unique opportunity for treatment. The risks of research were deemphasized, the benefits highlighted, and “fair access to research (both participation in research and access to the results of research) became as important as protection from exploitation” (Beauchamp and Childress 2001, p. 227). Concomitantly, growing number of research conducted in developing countries by researchers and sponsors from developed countries created a demand for involving host communities in collaborative partnership to avoid their exploitation in research and to ensure their just share in research benefits (cf. Emanuel and Grady 2006).

That shift towards justice considerations was reflected in the 2000 version of the DoH, especially in paragraph 30 (requiring the post-trial access to the best proven method identified by the study for every patient-subject; eventually significantly weakened by the second “Note of Clarification” adopted in 2004) and paragraph 19 (requiring study benefits to accrue also to the host populations). Nevertheless, justice-based concerns remained of secondary importance for the drafters of the 2000 DoH. The document maintained its predominant focus on the individual researcher’s duties derived from the principles of beneficence and of respect for autonomy. And—to a large extant—it ignored problems of justice and injustice in research practice (i.e., questions of: fair selection of research subjects and host communities, just distribution of research risks and benefits between individuals and communities, fair access to research and its results, fair benefit sharing).

The shift towards justice considerations found, however, strong expression in the third version of the International Ethical Guidelines for Biomedical Research Involving Human Subjects adopted by the Council for International Organizations of Medical Sciences in 2002. The Guidelines were built upon ethical standards laid down by the DoH and the Belmont Report (National Commission for the Protection of Human Subjects of Biomedical and Behavioral Research 1979), but they paid special attention to ethical challenges of research involving vulnerable individuals or communities as well as to dilemmas posed by multinational or transnational research conducted in low-income countries. Guideline 1 clearly stated that the production of socially valuable knowledge is “ethically justifiable only if it is carried out in ways that respect and protect, and are *fair* to, the subjects of that research and are morally acceptable within the communities in which the research is carried out” (CIOMS 2002). This has been repeated and developed further in the current version of the CIOMS Guidelines of 2016. The commentary on the 2016 Guideline 1 stresses that the respect for rights and welfare of individual participants and communities in which research is carried out, requires research to be (among others) “sensitive to issues of justice and fairness. This concern is manifest in choosing whose health needs are investigated; how risks, burdens, and anticipated benefits of individual studies are distributed; and who will have access to any resulting knowledge and interventions”.

Although specific demands of justice in research are not always evident or uncontroversial, all modern ethical standards for biomedical research include numerous, widely accepted requirements regarding the fair treatment of potential and actual research participants and relevant communities. A comprehensive discussion of these requirements lies beyond the scope of this article. One general imperative is however certain: research subjects’ interests should be protected against non-equal consideration and/or unfair treatment, especially when such a treatment aims at promoting interests of others (e.g., interests of researchers in minimizing inconveniences and maximizing efficacy and money-saving). This constitutes a justice-driven facet of the PP.

### Under the umbrella of human dignity and human rights discourse

One more paradigm shift occurred towards the end of twentieth century—the practice of medicine and medical research got embraced by the international human rights discourse based on the concept of inherent and unalienable human dignity (Thomasma 2001; Knowles 2001; Beyleveld and Brownsword 2001; Schroeder 2005; Andorno 2009, 2013). The recourse to the human dignity and human rights in the field of global bioethics was particularly strong in regulatory instruments developed under the auspices of the United Nations Educational, Scientific, and Cultural Organization (UNESCO) and the Council of Europe.

In 1997, after five years of drafting process, the Council of Europe adopted the Convention for the protection of human rights and dignity of the human being with regard to the application of biology and medicine: Convention on human rights and biomedicine, also known as the Oviedo Convection, since it was opened for signature in Oviedo, Spain (1997a). Seven years later, the Council of Europe adopted the Additional Protocol to the Convention concerning biomedical research (2005a) to “address the ethical and legal issues raised by present or future scientific advances through the further development, in specific fields such as biomedical research, of the principles contained in the Convention” (Council of Europe 2005b, para.11). The Convention (with subsequently added further additional protocols) was the very first and still is the only international legally binding human rights instrument entirely dedicated to biomedicine.

Both the Oviedo Convention and the Additional Protocol concerning biomedical research aim at protecting “the dignity and identity of all human beings and guarantee everyone, without discrimination, respect for their integrity and other rights and fundamental freedoms with regard to the application of biology and medicine” (Council of Europe 1997a, Article 1), and specifically with regard to any biomedical research involving interventions on human beings (Council of Europe 2005a, Article 1). The Preamble to the Additional Protocol clearly states that biomedical research contrary to human dignity and human rights should never be carried out, and stresses that the paramount concern should be the protection of the human being participating in research, particularly those who are vulnerable.

Importantly for this analysis, both the Oviedo Convention and the Additional Protocol concerning biomedical research create a strong link between the value of human dignity—which “constitutes the essential value to be upheld …[as] it is at the basis of most of the values emphasized in the Convention” (Council of Europe 1997b, para. 9)—and the PP. In both documents the PP is expressed in the very first substantive article, immediately following introductory provisions. Both documents stipulate that the interests and welfare of the human being, especially of the human being participating in research, shall prevail over the sole interest of society or science (Council of Europe 1997a, Article 2; Council of Europe 2005a, Article 3). Although a more detailed explanation of the PP is absent, it is evident that the PP sets limits of freedom of biomedical research. The scientific freedom is valuable, but not absolute. It is limited by the participants’ welfare and interests as well as the concept of human dignity and fundamental rights, expressed by the Additional Protocol, the Convention and “by other legal provisions ensuring the protection of the human being” (Council of Europe 2005a, Article 4; Council of Europe 2005b, para. 23). The Explanatory Reports to the Additional Protocol and the Convention clarify further that both documents are “inspired by the principle of the primacy of the human being, and all [their] Articles must be interpreted in this light” (Council of Europe 1997b, para. 22; Council of Europe 2005b, para. 21). Thus, under the Council of Europe bioethical framework, the PP plays two main roles: it is a general rule aiming at protecting humans (research subjects) against being sacrificed on the altar of science, and the fundamental, interpretative rule stating: if a provision is open to more than one interpretation, the interpretation which serves the human being’s interests and welfare in the most effective way should prevail.

In 2005, the UNESCO adopted the Universal Declaration of Human Rights and Bioethics (2005). The Declaration is not the first, but definitely it is the most important instrument adopted by the UNESCO dealing with biomedicine from the human right perspective. Similar to the Oviedo Convention, the Declaration assigns a very central role to the notion of human dignity and human rights, and it makes a direct link between this notion and the PP. The PP is spelled out in Article 3 of the Declaration titled “Human dignity and human rights”. Paragraph 1 of Article 3 states: “Human dignity, human rights and fundamental freedoms are to be fully respected”, and paragraph 2 reads: “The interests and welfare of the individual should have priority over the sole interest of science or society”. Thus, it should come as no surprise that numerous commentators of the Declaration consider the PP to be a normative offspring of the idea of human dignity (Andorno 2009, 2013; cf. Parker 2010; Simonsen 2012). Robert Andorno claims: “The primacy of the human being over science is indeed a direct corollary of the principle of respect for human dignity and aims to emphasize two fundamental ideas. First, that science is not an end in itself but only a means for improving the welfare of individuals and society. Second, that people should not be reduced to mere instruments for the benefit of science” (Andorno 2009, p. 228).

Obviously, the two ideas mentioned by Andorno do not exhaust all that is to be said on the dignity-derived PP. In order to fully understand the normative content of this PP, it would be necessary to discuss the meaning of human dignity. For obvious reasons, this is beyond the scope of this paper. Nevertheless, despite the vagueness and ambiguity of the notion of human dignity, it seems safe to conclude that in the context of global bioethics this notion includes two conceptions: the conception of human dignity as empowerment, which emphasizes human rights, especially the right to pursue one’s autonomously chosen goals, and the conception of human dignity as constraint, which is more concerned with human duties. These duties comprise “not only a duty to respect the dignity of others but also a duty not to compromise our own dignity, as well as to act in a way that is compatible with respect for the vision of human dignity that gives a particular community its distinctive cultural identity” (Beyleveld and Brownsword 2001, p. 1). Thus, human dignity as constraint is based on a shared vision of what constitutes valuable human life and what is owed to the individual—to her intrinsic worthiness, integrity, and identity. These dignity-driven duties fuel the PP which aims at protecting interests of human being participating in research, even against autonomy of the individual concerned.

### Towards a “difference position”

The heaviest criticism against the similarity position was launched at the beginning of the twenty-first century by a group of influential American research ethicists (Franklin G. Miller and his collaborators) which questioned its ethical and practical validity (Miller and Brody 2003, 2007; Miller and Rosenstein 2003). They argued that the very idea of researchers having a therapeutic obligation to research participants rests on a fundamental confusion—a “’therapeutic misconception” concerning the ethics of biomedical research (Miller and Brody 2003, p. 20), and it should be rejected. Their claim was based on the following reasoning: (i) “the basic goal and nature of the activity determines the ethical standards that ought to apply” (22); (ii) ultimate goals of medical practice and medical research are entirely different—physicians are devoted to promote the best medical interest of their individual patients; researchers aim at producing generable knowledge to improve medical care for the future patients; (iii) the goals of medical practice and medical research are “logically incompatible”, since “it is impossible to maintain fidelity to doing what is best for patients in the context of RCT [randomized clinical trials] because these are not designed for, and may conflict with personal care” (25); therefore (iv) medical research should be governed by ethical standards appropriate for goals and nature of the practice, which are distinct from the principle of therapeutic beneficence (and non-maleficence).

Miller’s et al. extended their critique of the similar position further to the requirement of clinical equipoise which, given the inappropriateness of applying the principle of therapeutic beneficence to research practice, provided a “solution” to a non-problem. Consequently, they claimed that: “Clinical equipoise is neither necessary nor sufficient for ethically justifiable RCTs [randomized clinical trials]. The use of placebo controls when proven effective treatment exists violates clinical equipoise … Nevertheless, it is the unacceptable level of risk, not the violation of investigators’ alleged “therapeutic obligation”, that makes these trials unethical” (Miller and Brody 2003, p. 25).

Thus, Miller and colleagues argued for abandoning both the similarity position and the clinical equipoise, and for adopting instead a “difference position” to the ethics of biomedical research involving humans, which would not conflate the ethics of research with the ethics of therapeutic medicine. They considered the “non-exploitation framework”, developed by Ezekiel Emanuel, David Wendler, and Christe Grady (2000), to be a strong candidate for such an alternative framework for the ethics of biomedical research.

The “non-exploitation framework” aims at protecting research participants from exploitation by setting seven requirements for all clinical research: (1) social value; (2) scientific validity; (3) fair subject selection; (4) favorable risk–benefit ratio; (5) independent review; (6) informed consent; (7) respect for potential and enrolled subjects (by “permitting withdrawal from research; protecting privacy through confidentiality; informing subjects of newly discovered risks and benefits; informing subjects of results of clinical research; maintaining welfare of subjects”; Emanuel et al. 2000, p. 2703). These requirements are justified by the following ethical values and social commitments: reasonable use of scare resources (1, 2); avoidance of exploitation (1, 2, 4); justice as equal treatment, justice as fairness in distribution of research risks and benefits (3); beneficence and non-maleficence (4, 7); public accountability and minimization of influence of potential conflicts of interests (5); respect for the subject’s autonomy (6, 7). This framework rejects the idea of researchers having any therapeutic obligations towards participants. It “holds instead, that researchers have the somewhat weaker duty to non-exploitation” (London 2007, p. 102).

The “non-exploitation framework” does not involve the PP. Perhaps, it is due to the PP original affiliation with the already rejected principle of therapeutic beneficence. In this context, it is interesting to mention that Ezekiel Emanuel has developed a proposal for a revision of the current version of the DoH adopted in 2013. The PP contained in the 2013 DoH reads: “While the primary purpose of medical research is to generate new knowledge, this goal can never take precedence over the rights and interests of individual research subjects” (para. 8). Paragraph 9 adds: “It is the duty of physicians who are involved in medical research to protect the life, health, dignity, integrity, right to self-determination, privacy, and confidentiality of personal information of research subjects. The responsibility for the protection of research subjects must always rest with the physician or other health care professionals and never with the research subjects, even though they have given consent”. Emanuel proposed to replace both provisions by one paragraph stating: "Purpose of Ethical Principles Guiding Research: The primary purpose of ethical principles to guide research in biomedical and other sciences involving human participants is to promote socially valuable studies while minimizing exploitation, the unfair distribution of benefits and burdens of the conduct and results of research. It should minimize the likelihood that the life, well-being, health, self-determination, privacy, and confidentiality of the participants’ personal information, might be adversely affected and minimize the consequences if they are” (Emanuel 2013, Supplementary appendix).

## Conclusion: the PP as a threefold concept

Despite many changes in the ethical framework for biomedical research involving human subjects sketched above, the PP is still present in the majority of international ethical standards and legal regulations on human research. They have incorporated the PP (reduced to one provision), under direct or indirect influence of the DoH authority, and elevated it up to the status of one of the most fundamental principles in research ethics, or bioethics in general. Despite its prominent regulatory position, however, the PP remains an enigmatic principle. With few exceptions noted earlier, little attention has been paid so far in the literature to the PP’s original roots and functions as well as to the impact of recent changes in research ethics paradigms on the PP’s normative meaning.

As shown in “Regulatory and normative origins of the PP” and “Normative roles of the original PPs” of this paper, the PP originated in the context of a very specific normative framework built around the principle of therapeutic beneficence. In all pre-2000 versions of the DoH the PP served two distinct roles: first, it was a procedural rule, mainly addressed to research ethics committees, imposing an obligation to carefully identify and weight the interests of research subjects against the interests of science and society; and second, a substantive rule aiming at protecting participants of non-therapeutic research from instrumental treatment and unacceptable risk. And as discussed in “The PP and the shift in the ethical framework for biomedical research”, the turn of twentieth century witnessed important changes in the ethics of biomedical research involving humans, which irreversibly undermined the accuracy of the ethical paradigm of similarity position and changed the ethical landscape of human research by, among others, emphasizing the significance of considerations regarding human dignity and justice in research. Only few scholars have commented on conceptual and normative consequences of those changes for the PP’s meaning and roles, especially as a basic interpretative guideline and a substantive protective rule.

Perhaps, one of the most significant features of the PP is that during over 40 years of its presence in ethical guidelines and legal regulations on biomedical research involving humans, it did not undergo significant linguistic changes. There are small variations in the PP’s wording in between guidelines and regulations, stemming from the fact that each of these documents has its own history, institutional context, conceptual and normative framework, and is a product of complex political negotiations and uneasy compromises. But, notwithstanding these variations, the PP preserves its general form. And this suggests that there is a core normative meaning of the PP common for all its formulations, able to accommodate changes taking place in the ethics of human research.

Drawing from the historical and normative analysis presented above, this final section claims that the PP is not a “vacuous figure of speech”, but a threefold concept:

- *A fundamental rule of interpretation* If a provision of an ethical or legal regulation on biomedical research involving humans is open to more than one interpretation, the interpretation which protects interests of the research participant in the most effective way should prevail.
- *A rule of procedure* Whenever a process of assessing research risks and potential benefits is conducted by researchers, sponsors, research ethics committees or other entitled entities, potential negative and positive effects of the research on the participants’ interests must be given the first and the most serious consideration. The rule provides justification for obligations to: (i) carefully identify, quantify (if possible), and assess risks and expected benefits (if any) to the interests of participants; and (ii) to clearly describe and explain—in a research protocol and information for potential participants, as well as in a research ethics committee’s opinion of the research ethical acceptability—which of the subjects’ interests have been taken into account, which risks and direct benefits have been analyzed, and how they have been assessed and weighed against the interests of science and society.
- *A substantive rule* No matter how high the potential social value of research is and no matter how favorable risk–benefit profile of the research is, the research must not (i) expose participants’ interests to research risk without their free and informed consent (if they are able to provide it); and (ii) impose *unacceptable* risks and burdens on participants’ interests that are beyond their free disposal, either due to their inability to give free and informed consent (or meaningful assent), or due to the role-related duties of other involved parties (researchers, physicians, parents, members of research ethics committees, etc.), requirements of justice (equality and fairness), or other value commitments, especially those hidden under the concept of human dignity.

Admittedly, the proposed interpretation of the PP needs further elaboration, especially when it comes to the PP seen as a substantive rule. It goes without saying that an upper level of acceptable risks and burdens should be different in different types of research/interventions (beneficial *v.* non-beneficial) and in different populations of subjects (volunteers *v.* individuals unable to give consent). Nevertheless, what makes research risk unacceptable, even when they are proportional to the expected benefits and adequately minimized, remains an open question. It is not surprising then, that the following three issues still raise controversies in research ethics and practice. First, which ethical duties, concerns or value commitments do justify setting an upper limit on net-risks in research with subjects able to give consent? Second, how such a maximal risk threshold should be determined and by whom? Third, how to justify and define a maximal acceptable level of risk associated with non-beneficial research involving incompetent subjects, e.g., infants? An exploration of these matters must be left to another occasion.

## References

[CR1] Abadie Roberto (2010). The professional guinea pig: Big pharma and the risky world of human subjects.

[CR2] Ammann Odile (2020). Domestic courts and the interpretation of International Law.

[CR3] Andorno Roberto (2009). Human dignity and human rights as a common ground for a global bioethics. Journal of Medicine and Philosophy.

[CR4] Andorno Roberto (2013). The dual role of human dignity in bioethics. Medicine, Health Care and Philosophy.

[CR5] Angell Marcia (1997). The ethics of clinical research in the Third World. The New England Journal of Medicine.

[CR6] Annas George J, Grodin Michael A (1998). Human rights and maternal-fetal HIV transmission prevention trials in Africa. American Journal of Public Health.

[CR7] Ashcroft Richard (1999). Equipoise, knowledge and ethics in clinical research and practice. Bioethics.

[CR8] Barak, Aharon. 2007. *Purposive interpretation in law*. Princeton University Press.

[CR9] Beauchamp Tom L, Childress James F (2001). The principles of biomedical ethics.

[CR10] Beyleveld Deryck, Brownsword Roger (2001). Human dignity in bioethics and biolaw.

[CR11] Brannon, Valerie C. 2018. Statutory interpretation: theories, tools, and trends. *Congressional Research Service Report* R45153. https://crsreports.congress.gov/product/pdf/R/R45153. Accessed 18 Dec 2020.

[CR12] Carlson Robert V, Boyd Kenneth M, Webb David J (2004). The revision of the Declaration of Helsinki: Past, present and future. The British Journal of Clinical Pharmacology.

[CR13] Council for International Organizations of Medical Sciences (CIOMS). 2002. International ethical guidelines for biomedical research involving human subjects. Geneva. https://cioms.ch/wp-content/uploads/2016/08/International_Ethical_Guidelines_for_Biomedical_Research_Involving_Human_Subjects.pdf. Accessed 18 Dec 2020. 14983848

[CR14] Council for International Organizations of Medical Sciences (CIOMS). 2016. International ethical guidelines for health-related research involving humans. Geneva. https://cioms.ch/wp-content/uploads/2017/01/WEB-CIOMS-EthicalGuidelines.pdf. Accessed 18 Dec 2020. 40523065

[CR15] Council of Europe. 1997a. Convention for the protection of human rights and dignity of the human beings with regard to the application on biology and medicine. Convention on human rights and biomedicine. CETS 164. Oviedo, Spain. https://rm.coe.int/168007cf98. Accessed 18 Dec 2020.

[CR16] Council of Europe. 1997b. Explanatory Report to the Convention for the protection of human rights and dignity of the human beings with regard to the application on biology and medicine. Convention on human rights and biomedicine. https://rm.coe.int/16800ccde5. Accessed 18 Dec 2020.

[CR17] Council of Europe. 2005a. Additional Protocol to Convention on human rights and biomedicine concerning biomedical research. CETS 195. Strasbourg, France. https://rm.coe.int/168008371a. Accessed 18 Dec 2020.

[CR18] Council of Europe. 2005b. Explanatory Report to Additional Protocol to Convention on human rights and biomedicine concerning biomedical research. Strasbourg, France. https://rm.coe.int/16800d3810. Accessed 18 Dec 2020.

[CR19] Djulbegovic Benjamin (2007). Articulating and responding to uncertainties in clinical research. The Journal of Medicine and Philosophy.

[CR20] Ellenberg Susan S, Temple Robert (2000). Placebo-controlled trials and active-control trials in the evaluation of new treatments. Part 2: Practical issues and specific cases. Annals of Internal Medicine.

[CR21] Emanuel Ezekiel J, Wendler David, Grady Christe (2000). What makes clinical research ethical?. JAMA.

[CR22] Emanuel Ezekiel J, Miller Franklin G (2001). The ethics of placebo-controlled trials—a middle ground. New England Journal of Medicine.

[CR23] Emanuel Ezekiel J, Grady Christine (2006). Four paradigms of clinical research and research oversight. Cambridge Quarterly of Healthcare Ethics.

[CR24] Emanuel, Ezekiel J. 2013. Reconsidering the Declaration of Helsinki. *The Lancet* 381: 1532–1533. Supplementary Webappendix: A Revised Declaration of Helsinki. https://www.thelancet.com/cms/10.1016/S0140-6736(13)60970-8/attachment/48800261-a41c-476d-bdd2-439d53a2ef7d/mmc1.pdf. Accessed 18 Dec 2020. 10.1016/s0140-6736(13)60970-8 23650661

[CR25] European Union. 2001. Directive 2001/20/EC of the European Parliament and of the Council of 4 April 2001 on the approximation of the laws, regulations and administrative provisions of the Member States relating to the implementation of good clinical practice in the conduct of clinical trials on medicinal products for human use. 16276663

[CR26] European Union. 2014. Regulations (EU) No 536/2014 of the European Parliament and of the Council of 16 April 2014 on clinical trials on medicinal products for human use, and repealing Directive 2001/20/EC.

[CR27] Feinberg Joel (1984). The moral limits of the criminal law. Vol. I: Harm to others.

[CR28] Fletcher Guy (2016). The philosophy of well-being: An introduction.

[CR29] Fletcher Guy (2016). The Routledge handbook of the philosophy of well-being.

[CR30] Forster Heidi P, Emanuel Ezekiel J, Grady Chrsite (2001). The 2000 revision of the Declaration of Helsinki: A step forward or more confusion?. The Lancet.

[CR31] Freedman Benjamin (1987). Equipoise and the ethics of clinical research. New England Journal of Medicine.

[CR32] Freedman Benjamin, Weijer Charles, Glass Kathleen C (1996). Placebo orthodoxy in clinical research. I: Empirical and methodological myths. The Journal of Law, Medicine & Ethics.

[CR33] Freedman Benjamin, Glass Kathleen C, Weijer Charles (1996). Placebo orthodoxy in clinical research II: Ethical, legal, and regulatory myths. The Journal of Law, Medicine & Ethics.

[CR34] Gifford Fred (1995). Community-equipoise and the ethics of randomized clinical trials. Bioethics.

[CR35] Grove Tara L (2020). Which textualism?. Harvard Law Review.

[CR36] Helgesson Gert, Eriksson Stefan (2008). Against the principle that the individual shall have priority over science. Journal of Medical Ethics.

[CR37] Helgesson Gert, Eriksson Stefan (2011). The moral primacy of the human being: A reply to Parker. Journal of Medical Ethics.

[CR38] Hey Spencer Phillips, London Alex John, Weijer Charles, Rid Annette, Miller Franklin (2017). Is the concept of clinical equipoise still relevant to research?. The BMJ.

[CR39] Human Delon, Fluss Sev S (2001). The World Medical Association’s declaration of Helsinki: Historical and contemporary perspectives.

[CR40] International Conference on Harmonisation of Technical Requirements for Registration of Pharmaceuticals for Human Use. 2016. ICH Harmonised Guideline Integrated Addendum To ICH E6(R1): Guideline For Good Clinical Practice E6(R2). https://database.ich.org/sites/default/files/E6_R2_Addendum.pdf. Accessed 18 Dec 2020.

[CR41] Jansen Lynn A (2005). A closer look at the bad deal trial: Beyond clinical equipoise. The Hastings Center Report.

[CR42] Joffe Steven, Miller Franklin G (2012). Equipoise: Asking the right questions for clinical trial design. Nature Reviews Clinical Oncology.

[CR43] Karlawish Jason H.Y., Lantos John (1997). Community equipoise and the architecture of clinical esearch. Cambridge Quarterly of Healthcare Ethics.

[CR44] Kimmelman Jonathan (2012). The social function of clinical equipoise. Clinical Trials.

[CR45] Knowles Lori P (2001). The lingua franca of human rights and the rise of a global bioethic. Cambridge Quarterly of Healthcare Ethics.

[CR46] Kuroyanagi Tatsuo (2009). On the 2008 revision to the WMA Declaration of Helsinki. Japan Medical Association Journal.

[CR47] Levine Robert J (1979). Clarifying the concepts of research ethics. The Hastings Center Report.

[CR48] Lie RK, Emanuel Ezekiel, Grady Christe, Wendler D (2004). The standard of care debate: The Declaration of Helsinki versus the international consensus opinion. Journal of Medical Ethics.

[CR49] Litton Paul, Miller Franklin G (2005). A normative justification for distinguishing the ethics of clinical research from the ethics of medical care. The Journal of Law, Medicine & Ethics.

[CR50] Litton Paul (2008). Non-beneficial pediatric research and the best interests standard: A legal and ethical reconciliation. Yale Journal of Health Policy, Law, and Ethics.

[CR51] Litton Paul (2012). A more persuasive justification for pediatric research. The American Journal of Bioethics.

[CR52] London Alex J, Steinbock B (2007). Clinical equipoise: Foundational requirement or fundamental error. The Oxford handbook to bioethics.

[CR53] Paweł Łuków, Różyńska Joanna, ten Have H (2015). Respect for autonomy. Encyclopedia of global bioethics.

[CR54] Lurie Peter, Wolfe Sidney M (1997). Unethical trials of interventions to reduce perinatal transmission of the human immunodeficiency virus in developing countries. The New England Journal of Medicine.

[CR55] Miller Franklin G, Brody Howard (2003). A critique of clinical equipoise: Therapeutic misconception in the ethics of clinical trials. The Hastings Center Report.

[CR56] Miller Franklin G, Rosenstein Donald L (2003). The therapeutic orientation to clinical trials. The New England Journal of Medicine.

[CR57] Miller Franklin G, Brody Howard (2007). Clinical equipoise and the incoherence of research ethics. The Journal of Medicine and Philosophy.

[CR58] Miller Paul B, Weijer Charles (2003). Rehabilitating equipoise. The Kennedy Institute of Ethics Journal.

[CR59] Miller Paul B, Weijer Charles (2006). Fiduciary obligation in clinical research. The Journal of Law, Medicine & Ethics.

[CR60] Miller Paul B, Weijer Charles (2006). Trust based obligations of the state and physician-researchers to patient-subjects. Journal of Medical Ethics.

[CR61] Miller Paul B, Weijer Charles (2007). Equipoise and the duty of care in clinical research: A philosophical response to our critics. The Journal of Medicine and Philosophy.

[CR62] Millum Joseph, Wendler David, Emanuel Ezekiel (2013). The 50th anniversary of the Declaration of Helsinki: Progress but many remaining challenges. JAMA.

[CR63] Morris Kelly (2013). Revising the Declaration of Helsinki. The Lancet.

[CR64] National Commission for the Protection of Human Subjects of Biomedical and Behavioral Research. 1979. The Belmont report: Ethical principles and guidelines for the protection of human subjects of research. U.S. Department of Health and Human Services. https://www.hhs.gov/ohrp/regulations-and-policy/belmont-report/read-the-belmont-report/index.html. Accessed 18 Dec 2020. 25951677

[CR65] Nelson Caleb (2005). What is textualism?. Virginia Law Review.

[CR66] Parker Colin (2010). The moral primacy of the human being. Journal of Medical Ethics.

[CR67] Pattinson Shaun D (2012). Emergency research and the interests of participants. Medical Law International.

[CR68] Piasecki Jan, Waligora Marcin, Dranseika Vilius (2015). Non-beneficial pediatric research: Individual and social interests. Medicine, Health Care and Philosophy.

[CR69] Riis Povl (2003). Thirty years of bioethics: The Helsinki Declaration 1964–2003. New Review of Bioethics.

[CR70] Rothman Kenneth J, Michels Karin B (1994). The continuing unethical use of placebo controls. The New England Journal of Medicine.

[CR71] Różyńska Joanna (2015). On the alleged right to participate in high-risk research. Bioethics.

[CR72] Schroeder Doris (2005). Human rights and their role in global bioethics. Cambridge Quarterly of Healthcare Ethics.

[CR73] Shah Seema (2013). Does research with children violate the best interests standard? An empirical and conceptual analysis. Northwestern Journal of Law and Social Policy.

[CR74] Shamoo Adil E (2008). The myth of equipoise in phase 1 clinical trials. The Medscape Journal of Medicine.

[CR75] Simonsen Sigmund (2012). Acceptable risk in biomedical research: European perspectives.

[CR76] Sumner, Leonard W. 1996. *Welfare, Happiness, and Ethics*. Oxford University Press.

[CR77] Temple Robert, Ellenberg Susan S (2000). Placebo-controlled trials and active-control trials in the evaluation of new treatments. Part 1: ethical and scientific issues. Annals of Internal Medicine.

[CR78] Thomasma David (2001). Proposing a new agenda: Bioethics and international human rights. Cambridge Quarterly of Healthcare Ethics.

[CR79] United Nations Educational, Scientific and Cultural Organization (UNESCO). 2005. Universal Declaration on bioethics and human rights. http://portal.unesco.org/en/ev.php-URL_ID=31058%26URL_DO=DO_TOPIC%26URL_SECTION=201.html. Accessed 18 Dec 2020.

[CR80] Veatch Robert M (2002). Indifference of subjects: An alternative to equipoise in randomized clinical trials. Social Philosophy & Policy.

[CR81] Veatch Robert M (2007). The irrelevance of equipoise. The Journal of Medicine and Philosophy.

[CR82] Waligóra Marcin, Strzebońska Karolina, Wasylewski Mateusz T (2018). Neither the harm principle nor the best interest standard should be applied to pediatric research. The American Journal of Bioethics.

[CR83] World Health Organization (WHO) (1995). Guidelines for good clinical practice (GCP) for trials on pharmaceutical products.

[CR84] World Medical Association (WMA). 1975. Declaration of Helsinki. Recommendations guiding medical doctors in biomedical research involving human subjects. Tokyo, Japan. https://www.wma.net/wp-content/uploads/2018/07/DoH-Oct1975.pdf. Accessed 18 Dec 2020.

[CR85] World Medical Association (WMA). 1989. Declaration of Helsinki. Recommendations guiding medical doctors in biomedical research involving human subjects. Hong Kong. https://www.wma.net/wp-content/uploads/2018/07/DoH-Sept1989.pdf. Accessed 18 Dec 2020.

[CR86] World Medical Association (WMA). 2000. Declaration of Helsinki. Ethical principles for medical research involving human subjects. Edinburgh, Scotland. https://www.wma.net/wp-content/uploads/2018/07/DoH-Oct2000.pdf. Accessed 18 Dec 2020.

[CR87] World Medical Association (WMA). 2008. Declaration of Helsinki. Ethical Principles for Medical Research Involving Human Subjects. Seoul, Korea. https://www.wma.net/wp-content/uploads/2018/07/DoH-Oct2008.pdf. Accessed 18 Dec 2020.

[CR88] World Medical Association (WMA). 2013. Declaration of Helsinki. Ethical Principles for Medical Research Involving Human Subjects. Fortaleza, Brazil. https://www.wma.net/policies-post/wma-declaration-of-helsinki-ethical-principles-for-medical-research-involving-human-subjects/. Accessed 18 Dec 2020.

[CR89] Zarbiyev Fuad, Bianchi Andrea, Peat Daniel, Windsor Matthew (2015). A genealogy of textualism in treaty interpretation. Interpretation in international law.

